# Relationship between Oxidative Stress and Physical Activity in Women with Squamous Intraepithelial Lesions in a Cervical Cancer Control Program in the Brazilian Amazon

**DOI:** 10.1155/2019/8909852

**Published:** 2019-10-14

**Authors:** Saul Rassy Carneiro, Abner Ariel da Silva Lima, Gleyce de Fátima Silva Santos, Cláudia Simone Baltazar de Oliveira, Maria Cláudia Valente Almeida, Maria da Conceição Nascimento Pinheiro

**Affiliations:** ^1^Hospital Universitário João de Barros Barreto, Núcleo de Medicina Tropical, Universidade Federal do Pará, Brazil; ^2^Núcleo de Medicina Tropical, Universidade Federal do Pará, Brazil; ^3^Faculdade de Fisioterapia e Terapia Ocupacional, Universidade Federal do Pará, Brazil; ^4^Laboratório de Estresse Oxidativo, Núcleo de Medicina Tropical, Universidade Federal do Pará, Brazil

## Abstract

Human papillomavirus (HPV) infection is recognized as the most common sexually transmitted disease in the world, and there is a consensus on its role in the etiology of preneoplastic epithelial changes in the cervix. Through the process of lipid peroxidation, oxidative stress is found in the course of premalignant and malignant changes. Moreover, the level of physical activity can exert an influence on markers of oxidative stress, lowering the serum levels of these markers. *Objective*. To determine the relationship between levels of malondialdehyde (MDA) and the level of physical activity in women with squamous intraepithelial lesion (SIL) of the cervix. *Methods*. A cross-sectional study was conducted with 46 women participating in a cervical cancer control program. The women had been submitted to the cytopathological exam and were divided into two groups: 18 with SIL and 28 controls. MDA concentrations were determined, and the International Physical Activity Questionnaire (IPAQ) was administered on the same day as the gynecological appointment (prior to the Papanicolaou test). *Results*. The SIL group had higher MDA levels than the control group (mean: 47.63 ± 9.57 vs. 9.32 ± 4.79, respectively) and a lower IPAQ score (median: 713.5 vs. 1875, respectively). A weak correlation was found between the MDA level and IPAQ score (*r*^2^ = −0.34, *p* = 0.018). *Conclusion*. The women with SIL had higher levels of oxidative stress and were less physically active than the women in the control group. These findings suggest that physical exercise exerts an influence on markers of oxidative stress in the development of intraepithelial squamous lesions.

## 1. Introduction

Squamous intraepithelial lesions (SIL) are precursors to cervical cancer. The central agent in the pathogenesis of SIL is human papillomavirus (HPV), which is detected in 97% of cases [1, 2]. Although preventable and with a good prognosis, HPV infection and SIL affect 20 to 60% of the female population and can evolve to squamous cell carcinoma of the cervix, especially the malignant type, in women of the Amazon region in Brazil [3].

The role of oxidative stress in the pathogenesis of cancer is widely discussed. The chronic presence of reactive oxygen species (ROS) is thought to favor the integration of the viral oncogene with cellular DNA, leading to the overexpression of proteins HR, E6, and E7 and the consequent formation of tumor cells. Malondialdehyde (MDA) alone is an important cellular mutagenic agent [4, 5]. A direct association has been found between lipid peroxidation expressed by MDA and cervical cancer as well as precursor squamous lesions, although few studies have specifically addressed SIL [6, 7].

The practice of physical activity is considered an important factor in the prevention of diseases, probably due to the improvement in antioxidant defenses [7]. However, little is known regarding the relationship between squamous lesions and the level of physical activity among affected women, as no studies have related these variables.

There are several studies indicating the increased formation of serum oxidative markers in patients with cervical cancer; some have shown that even premalignant lesions may regulate oxidative stress but there is no research indicating that physical activity may be associated with the reduction of serum levels of lipid peroxidation in women with SIL [4–7]. This may indicate a new approach to recommendation and intervention based on regular physical activity in the treatment of all stages of lesions.

Therefore, the aim of the present study was to analyze the association between serum levels of MDA and the level of physical activity in women with SIL at a cervical cancer prevention and control service in the Amazon region of Brazil.

## 2. Materials and Methods

### 2.1. Patients and Study Design

A cross-sectional study was conducted with women at the Cervical Cancer Prevention Service of the Center for Tropical Medicine of the Universidade Federal do Pará, Brazil. This service meets the spontaneous demand for care from users of the Brazilian public health system sent from primary care units or through direct referrals from the university as well as from research and extension programs of the university.

All women who underwent the Papanicolaou test at the service between February 20 and June 27, 2018, were asked to participate in the study. Those who met the following inclusion criteria were selected to compose the sample: age 18 years or older and agreement to participate in all steps of the study. Women with cognitive limitations, a diagnosis or undergoing treatment for cancer of any type, innate or acquired immunodeficiency, and making regular use of a corticosteroid and those with autoimmune or infectious-parasitic diseases were excluded from the study.

Eighteen women formed the group with SIL. Twenty-eight women matched for age with the SIL group (±2 years) with a cytopathological exam considered normal or with atypical squamous cells of an undetermined significance (ASCUS) [8] composed the control group. The cervicovaginal material collected and processed for the Papanicolaou test was submitted to cytopathological analysis based on the Bethesda system by experts in the field in accordance with the guidelines of the Brazilian Health Ministry for screening for cervical cancer [8, 9].

### 2.2. Epidemiological Data

Epidemiological data and reproductive information were acquired from the medical chart of each patient: age, marital status, schooling, number of children, smoking, eating habits, and self-perceived states of stress and/or anxiety at work or home. Information not found on the charts was obtained directly from the patients on the day of the gynecological exam.

### 2.3. Level of Physical Activity

Prior to the collection of the material for the cytopathological exam, the patients answered the short form of the International Physical Activity Questionnaire (IPAQ). This questionnaire was developed to estimate the level of physical activity among adults between 18 and 65 years of age, but studies have demonstrated its applicability to elderly individuals up to 90 years of age [10]. The IPAQ was designed by a workgroup that involved 14 research centers from 12 countries. Data on its reliability and applicability were published in 2003. The questionnaire is composed of eight items addressing the time spent per week walking, daily activities that involve moderate or intense effort, and time spent on sedentary activities [11]. The questions are listed below:

1. During the last 7 days, on how many days did you walk for at least 10 minutes at a time?
2. How much time did you usually spend walking on one of those days?
3. During the last 7 days, on how many days did you perform moderate activities for at least 10 continuous minutes?
4. How much total time per day did you spend on these activities?
5. During the last 7 days, on how many days did you perform vigorous activities for at least 10 continuous minutes?
6. How much total time per day did you spend on these activities?
7. How much total time did you spend in a sitting position on a weekday?
8. How much total time did you spend in a sitting position on a weekend day?

The answers were entered into a Microsoft Excel® program. Predefined formulas were used to calculate the number of calories spent during a week. The respondents were then categorized as sedentary, mildly active, moderately active, or highly active.

### 2.4. Determination of Serum MDA

**Figure 1:**
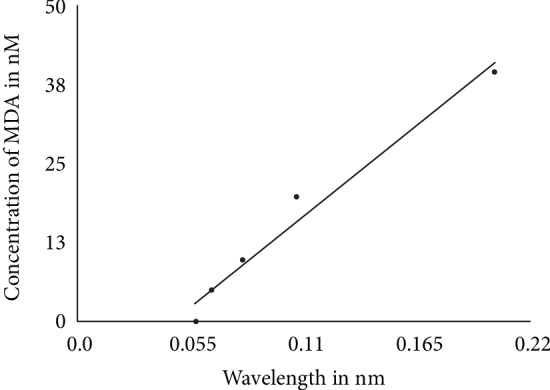
Linear regression curve of standard concentration of MDA (0, 5, 10, 20, and 40 nM) with a correlation coefficient of 0.972 and regression equation of *y* = 264.5*x* − 11.979.

Blood samples were collected from the vein of the forearm using a vacutainer and stored in test tubes with anticoagulant and EDTA. The serum was then separated from the plasma for the biochemical analysis. Serum MDA was analyzed by the reaction between MDA and thiobarbituric acid (TBA) in low pH and a high temperature to form the MDA-TBA complex, which has a pinkish color and maximum absorption at 535 nm. Lipid peroxidation was measured by estimating thiobarbituric acid reactive substances (TBARS). The method consisted of the precipitation of the lipoproteins of the samples by the addition of 1% trichloroacetic acid, 1% TBA, and sodium hydroxide. The union of lipid peroxide and TBA was performed by heating in a water bath for 30 minutes. The formed chromogens were extracted in n-butanol and read at 535 nm. Lipid peroxidation was expressed as nmol/ml of MDA (Isaksson et al., 2009). The calculation was performed using a five-point calibration curve (0, 5, 10, 20, and 40 nM) established from an MDA solution (tetra-hydroxypropane) of 20 nM [12] (Figure 1).

### 2.5. Statistical Analysis

The data were represented in graphs and tables and analyzed using descriptive statistics (absolute and percentage frequency, mean and standard deviation, and median and interquartile range (25^th^ to 75^th^ percentile)). The D'Agostino test was used to determine the distribution (normal or nonnormal) of the data. Depending on the result, either Student's *t*-test or the Mann-Whitney test was used to compare continuous variables between groups. The chi-square test and Fisher's exact test were employed to compare nominal and categorical variables. Spearman's correlation coefficient was calculated to determine the strength of the correlation between serum MDA and the IPAQ score. All statistical tests were performed with the aid of SPSS version 20.0, with the level of significance set to 5% (*p* < 0.05).

### 2.6. Ethical Aspects

This study received approval from the Human Research Ethics Committee of the Center for Tropical Medicine of the Universidade Federal do Pará (certificate number 2.051.391) and was conducted in accordance with the determinations of Resolution 466/2012 of the Brazilian National Board of Health. All volunteers agreed to participate by signing a statement of informed consent.

## 3. Results

### 3.1. Description of the Groups

During the entire study period, a total of 163 women visited the clinic of the Cervical Cancer Prevention Service; 20 of whom had SIL of the cervix and the rest had either normal results or ASCUS. A problem occurred during the blood collection from two patients in the SIL group. Therefore, these women were excluded from the study, and the case group was composed of 18 patients. The control group was formed using a randomization process: simple lottery with sealed envelopes containing the registration number of patients in the target age range (±2 years) in comparison to the patients in the case group. Thus, 28 age-matched women whose cytopathological exam results were either normal or ASCUS composed the control group.

Mean age was 52.17 years in the case group and 47.96 years in the control group. Chronic noncommunicable diseases were reported in 61% of the case group and 50% of the control group. Smoking was reported in 17.9% of the control group and none of the patients in the SIL group. Self-reported food restriction or dieting was found in 22.2% of the case group and 28.6% of the control group. A total of 61.1% of the women in the SIL group and 39.3% of the women in the control group had more than two children. A total of 50% of the SIL group and 42.9% of the control group reported being either married or in a stable union. Reports of stress in the work or home setting were found in 71.8% of the SIL group and 60.7% of the control group (Table 1). Mean blood sugar was 98.72 mg/dL in the SIL group and 104.32 mg/dL in the control group. Triglyceride levels were 165 mg/mL and 165.19 mg/mL, respectively. Total cholesterol and fraction (HDL, VLDL, and LDL) levels were, respectively, 200, 58, 33, and 110 mg/dL in the SIL group and 196, 61, 30, and 102 mg/dL in the control group. Transaminases (TGO and TGP) were, respectively, 31 and 34 mg/dL in the SIL group and 27 and 31 mg/dL in the control group (Table 2).

### 3.2. Serum Levels of TBARS and IPAQ Score

**Figure 2:**
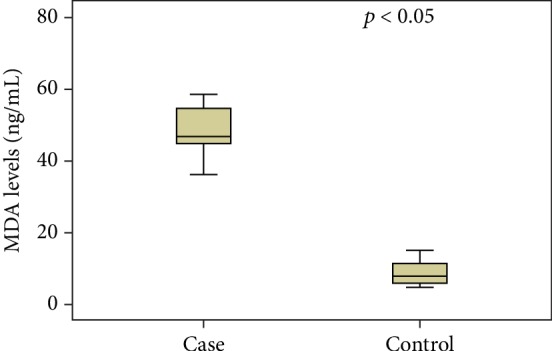
Comparison of serum MDA levels between groups.

**Figure 3:**
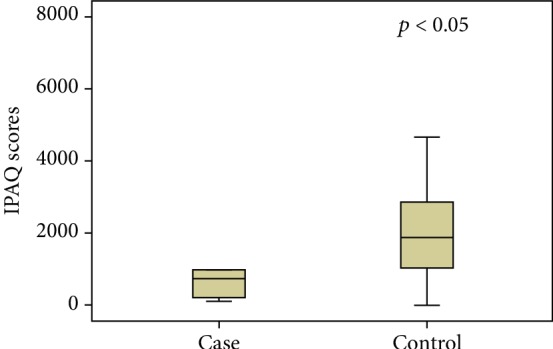
Comparison of IPAQ scores between groups.

**Figure 4:**
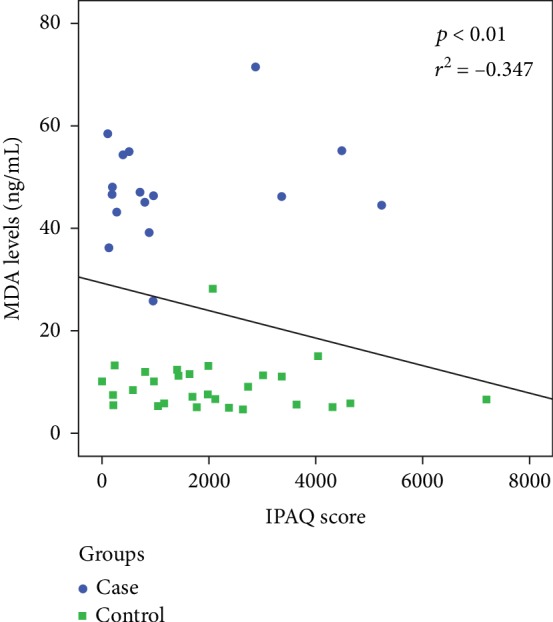
Correlation between MDA levels and IPAQ scores.

A difference was found in the serum MDA concentration between the groups (Figure 2). The women in the SIL group had significantly higher levels of lipid peroxidation compared to those whose test results were normal or revealed nonspecific inflammatory lesions. The IPAQ score was lower in the SIL group, charactering less physical activity in comparison to the control group (Figure 3). A weak, inversely proportional correlation was found between the MDA levels and IPAQ scores (Figure 4), suggesting that the level of physical activity may exert an influence on serum MDA concentrations and consequently affect the oxidative balance of the organism.

## 4. Discussion

Few studies have analyzed oxidative stress among individuals in cancer screening and prevention programs, and fewer still have correlated levels of oxidative stress in women with SIL with a physical activity assessment tool, such as the IPAQ. In the present study, women with SIL had higher levels of MDA than those with a normal cytopathological exam or ASCUS. Moreover, the women in the SIL group had lower IPAQ scores, characterizing less effort exerted on activities of daily living in comparison to the control group. Indeed, an inverse correlation was found between MDA levels and IPAQ scores.

Many studies have demonstrated that HPV infection is associated with an imbalance between the production of free radicals and the antioxidant response of the organism [13]. Camini and colleagues report that viral infections participate in processes that involve an increase in the production of ROS, which is due to phagocyte activation induced by inflammation stemming from the virus. The production of a large amount of ROS plays an important role in the transformation of the cells of the cervix, favoring the integration of viral oncogenes to cellular DNA [14].

Studies report a correlation between the production of ROS and cervical cancer as a concomitant factor and even an etiological factor for the development of the neoplastic process. However, few studies have focused on nonspecific and premalignant inflammatory lesions, the latter of which may be submitted to either a “wait-and-see” or therapeutic (excision of the affected region) approach, depending on the clinical criteria or results of the cytopathological exam [15, 16].

In the present study, the women with SIL had higher serum levels of MDA in comparison to those whose exam results were normal or revealed nonspecific inflammatory lesions. Gonçalves and colleagues demonstrated that TBARS levels were significantly higher in women with malignant and premalignant lesions compared to the control group and found a positive association between lipid peroxidation and the severity of the lesions; the authors also found that individuals with low-grade SIL also had higher levels than the control group [17]. Studying 202 samples from colpo-cytopathological exams, Visalli et al. also found that the levels of oxidative stress in patients with more severe SIL were much higher than the levels in the control group and a significant increase was found even among those with low-grade SIL [18].

Questionnaires addressing physical activity can be valuable, and low-cost tools for estimating the degree of physical activity in a sample exhibit good correlations when compared to methods that use physiological markers or movement meters [19, 20]. In the present study, the women with the SIL had lower IPAQ scores compared to the control group, which could mean that the women in the control group were more physically active. Some studies have found that the level of physical activity has a direct impact on the health status of a population and should be part of a nonpharmacological approach to diverse pathological processes [21, 22]. Bauman et al. demonstrated the viability of the administration of the IPAQ in a population of 49,493 individuals from 20 countries. The authors found satisfactory results regarding the classification of the physical activity level of the participants [23], providing further evidence of the usefulness of this simple, accessible, adaptable assessment tool in studies involving heterogeneous samples of patients [23].

An inverse correlation was found between levels of oxidative stress based on serum MDA and the IPAQ score. As all other variables studied were similar between the two groups, this correlation suggests the influence of the quantity and quality of energy expenditure during daily activities on lipid peroxidation.

Analyzing 53 healthy individuals submitted to different exercise programs, Bouzid et al. demonstrated that lipid peroxidation levels were lower among those that adhered to the program in comparison to the control group, which had higher levels of serum TBARS [24]. Likewise, Vincent et al. conducted a study involving 49 obese older adults submitted to regular physical exercise for six months and found that TBARS levels were significantly lower at the end of the program [25]. However, some authors report that the reduction in oxidative stress depends on the intensity of the exercise, with exercises of mild to moderate intensity offering the most benefit [26–28].

There are different biochemical pathways regarding the production of ROS related to physical activity, and different types and levels of activity elicit different organic responses. Therefore, one must consider the different effects of aerobic exercises and activities that predominantly involve the anaerobic metabolism as well as the intensity at which each exercise is performed. Although not fully understood, the adaptive response to exercise demonstrates a clear relationship to such particularities [29].

The present study has limitations that should be considered. Only one marker of oxidative stress was used: serum MDA through the TBARS method involving spectrophotometry. It is possible that more substantial results would have been obtained if other oxidant and even antioxidant markers had been used for a more accurate comparison of the REDOX equilibrium. Another limitation was not having better control over variables regarding the daily lives of the participants, as the analysis was limited to self-reported data on physical activity, eating habits, lifestyle habits, smoking, and alcohol intake. Finally, the sample size was small due to the detection of diagnosed cases during the study.

In conclusion, the present investigation is a pioneering study on the association between physical activity level and oxidative stress in women participating in a cervical cancer prevention program. The findings reveal that women with squamous intraepithelial lesions had higher levels of serum MDA and lower scores on the International Physical Activity Questionnaire in comparison to the control group and an inverse correlation was found between these two variables in the overall sample. These findings pave the way for further studies on the influence of different levels of physical activity on the oxidative response in women with squamous intraepithelial lesions of the cervix, offering novel options regarding prevention, therapy, and health promotion actions for these patients.

## Figures and Tables

**Table 1:** Epidemiological, reproductive, and social characteristics and self-reported diseases.

Variable	SIL group (*n* = 18)	Control group (*n* = 28)	*p* value
Age	Mean: 52.17 ± 15.15	Mean: 47.96 ± 14.36	NS^∗^
Schooling			NS^∗∗^
≤8 years of study	75%	38.5%	
>8 years of study	25%	61.5%	
Declared income			NS^∗∗^
≤BMMW	91.7%	84.6%	
2‐3 × BMMW	8.3%	15.4%	
Number of children			NS^∗∗^
None	11.1%	14.3%	
≤2	27.8%	46.4%	
>2	61.1%	39.3%	
Self-reported stress at work or home			NS^∗∗^
Yes	71.8%	60.7%	
No	22.2%	39.3%	
Chronic noncommunicable disease			NS^∗∗^
Yes	61.1%	50.0%	
No	38.9%	50.0%	
Reported dieting or food restriction			NS^∗∗^
Yes	22.2%	28.6%	
No	77.8%	71.4%	
Smoking			NS^∗∗^
Yes	0.0%	17.9%	
No	100%	82.1%	
Marital status			NS^∗∗^
Single or separated	50.0%	57.1%	
Married or in stable union	50.0%	42.9%	

Source: Cervical Cancer Prevention Service, Center for Tropical Medicine. NS: nonsignificant; BMMW: Brazilian monthly minimum wage. ^∗∗^Chi-square test. ^∗^Student's *t*-test.

**Table 2:** Biochemical characteristics of participants.

Variable	SIL group Mean ± SD (mg/dL)	Control group Mean ± SD (mg/dL)	*p* value
Total cholesterol total	200 ± 30	196 ± 49	NS
HDL	58 ± 12	61 ± 18	NS
LDL	110 ± 27	102 ± 34	NS
VLDL	33 ± 17	30 ± 19	NS
TGO	31 ± 33	27 ± 10	NS
TGP	34 ± 51	31 ± 14	NS
Blood sugar	98.72 ± 18.32	104.32 ± 51.78	NS
Triglycerides	165 ± 88	165.19 ± 117.5	NS

Source: Oxidative Stress Laboratory (NMT) and Clinical Analysis Laboratory (ICB). NS: nonsignificant. Student's *t*-test.

## Data Availability

The data used to support the findings of this study are available from the corresponding author upon request.

## Supplementary Materials

Supplementary Materials In the file, there are the score of IPAQ, the values of all the variables shown in Table 2, and the MDA concentration in both groups (caso in Portuguese as the case group in English and controle as the control group).
