# Supplementary material for: Relationship between Oxidative Stress and Physical Activity in Women with Squamous Intraepithelial Lesions in a Cervical Cancer Control Program in the Brazilian Amazon
Source: Oxid Med Cell Longev. 2019 Oct 14;2019:8909852. doi: 10.1155/2019/8909852 (PMC6815630; doi:10.1155/2019/8909852)
Supplement: Supplementary Materials — In the file, there are the score of IPAQ, the values of all the variables shown in Table 2, and the MDA concentration in both groups (caso in Portuguese as the case group in English and controle as the control group). [file 8909852.f1.pdf]

Participant ID	blood sugar	MDA	IPAQ-SF Responses (ENTER RAW DATA HERE) Vigorous Activity Days Q1	Vigorous Activity Min Q2	Moderate Activity Days Q3	Moderate Activity Min Q4	Walking Activity Days Q5	Walking Activity Min Q6	Sitting Hours Q7	IPAQ-SF Intermediary Calculations Vigorous (min/d) (Q2 Truncated)	Moderate (min/d) (Q4 Truncated)	Walking (min/d) (Q6 Truncated)	Categorical Score Criteria: High Activity Criteria ≥3 Days Vig (≥1500 MET-min/wk)	High Activity Criteria ≥7 Days Any (≥3000 MET-min/wk)	Moderate Activity Criteria ≥3 Days Vig (≥20 min/d)	Moderate Activity Criteria ≥5 Days Mod/Walk (≥30 min/d)	Moderate Activity Criteria ≥5 Days Any (≥600 MET-min/wk)	IPAQ-SF Final MET-Minutes and Categorical Scores: TOTAL DAYS OF ACTIVITY Days	TOTAL DAYS OF ACTIVITY Days (Recoded)	TOTAL ACTIVITY (min/wk) (Self-Reported)	TOTAL ACTIVITY (min/wk) (Truncated)	MET-MINUTES PER WEEK Vigorous	MET-MINUTES PER WEEK Moderate	MET-MINUTES PER WEEK Walk	MET-MINUTES PER WEEK Total	PHYSICAL ACTIVITY CATEGORY Category	PHYSICAL ACTIVITY CATEGORY Category (Recoded)	OUTLIER (Self-Reported Total Activity >960 min)
1	85	0.563	0	0	1	240	0	0	4	0	180	0	N	N	N	N	N	1	1	240	180	0	720	0	720	Low	1	N
2	94	0.822	2	540	0	0	0	0	1	180	0	0	N	N	N	N	N	2	2	540	180	2880	0	0	2880	Low	1	N
3	99	0.626	1	30	1	15	1	30	3	30	15	30	N	N	N	N	N	3	3	75	75	240	60	99	399	Low	1	N
4	99	0.074	0	0	0	0	1	60	4	0	0	60	N	N	N	N	N	1	1	60	60	0	0	198	198	Low	1	N
5	77	0.536	0	0	1	20	1	30	2	0	20	30	N	N	N	N	N	2	2	50	50	0	80	99	179	Low	1	N
6	86	0.116	5	720	0	0	0	0	2	180	0	0	Y	N	Y	N	Y	5	5	720	180	7200	0	0	7200	High	3	N
7	93	0.673	0	0	0	0	2	15	8	0	0	15	N	N	N	N	N	2	2	15	15	0	0	99	99	Low	1	N
8	97	0.552	0	0	0	0	2	30	2	0	0	30	N	N	N	N	N	2	2	30	30	0	0	198	198	Low	1	N
9	90	0.060	1	300	2	60	2	30	1	180	60	30	N	N	N	N	Y	5	5	390	270	1440	480	198	2118	Moderate	2	N
10	110	0.066	1	240	0	0	0	0	5	180	0	0	N	N	N	N	N	1	1	240	180	1440	0	0	1440	Low	1	N
11	91	0.134	0	0	2	180	2	210	2	0	180	180	N	N	N	N	N	4	4	390	360	0	1440	1188	2628	Low	1	N
12	90	0.141	1	120	7	120	0	0	1	120	120	0	N	Y	N	Y	Y	8	7	240	240	960	3360	0	4320	High	3	N
13	110	0.534	0	0	2	120	0	0	1	0	120	0	N	N	N	N	N	2	2	120	120	0	960	0	960	Low	1	N
14	98	0.451	2	30	0	0	4	30	6	30	0	30	N	N	N	N	Y	6	6	60	60	480	0	396	876	Moderate	2	N
15	89	0.520	0	0	0	0	2	120	2	0	0	120	N	N	N	N	N	2	2	120	120	0	0	792	792	Low	1	N
16	90	0.635	3	360	0	0	2	25	2	180	0	25	Y	N	Y	N	Y	5	5	385	205	4320	0	165	4485	High	3	N
17	98	0.173	1	540	1	60	7	30	5	180	60	30	N	N	N	Y	Y	9	7	630	270	1440	240	693	2373	Moderate	2	N
18	219	0.151	0	0	7	30	3	20	2	0	30	20	N	N	N	Y	Y	10	7	50	50	0	840	198	1038	Moderate	2	N
19	99	0.118	0	0	7	30	7	50	9	0	30	50	N	N	N	Y	Y	14	7	80	80	0	840	1155	1995	Moderate	2	N
20	96	0.138	0	0	0	0	6	40	9	0	0	40	N	N	N	Y	Y	6	6	40	40	0	0	792	792	Moderate	2	N
21	97	0.082	0	0	0	0	0	0	5	0	0	0	N	N	N	N	N	0	0	0	0	0	0	0	0	Low	1	N
22	103	0.531	0	0	7	120	0	0	1	0	120	0	N	Y	N	Y	Y	7	7	120	120	0	3360	0	3360	High	3	N
23	109	0.187	2	840	0	0	2	20	6	180	0	20	N	N	N	N	N	4	4	860	200	2880	0	132	3012	Low	1	N
24	71	0.087	0	0	7	40	6	15	1	0	40	15	N	N	N	Y	Y	13	7	55	55	0	1120	297	1417	Moderate	2	N
25	91	0.128	0	0	2	150	7	150	5	0	150	150	N	Y	N	Y	Y	9	7	300	300	0	1200	3465	4665	High	3	N
26	86	0.130	0	0	1	120	2	15	4	0	120	15	N	N	N	N	N	3	3	135	135	0	480	99	579	Low	1	N
27	73	0.098	0	0	5	10	0	0	4	0	10	0	N	N	N	N	N	5	5	10	10	0	200	0	200	Low	1	N
28	76	0.076	0	0	0	0	7	10	3	0	0	10	N	N	N	N	N	7	7	10	10	0	0	231	231	Low	1	N
29	97	0.543	0	0	3	30	7	15	8	0	30	15	N	N	N	N	Y	10	7	45	45	0	360	347	707	Moderate	2	N
30	89	0.152	0	0	2	360	2	30	5	0	180	30	N	N	N	N	N	4	4	390	210	0	1440	198	1638	Low	1	N
31	81	0.068	0	0	0	0	5	120	1	0	0	120	N	N	N	Y	Y	5	5	120	120	0	0	1980	1980	Moderate	2	N
32	90	0.514	0	0	7	180	5	30	3	0	180	30	N	Y	N	Y	Y	12	7	210	210	0	5040	495	5535	High	3	N
33	328	0.077	1	120	2	180	5	20	3	120	180	20	N	N	N	N	Y	8	7	320	320	960	1440	330	2730	Moderate	2	N
34	80	0.133	5	15	3	30	2	30	3	15	30	30	N	N	N	Y	Y	10	7	75	75	600	360	198	1158	Moderate	2	N
35	76	0.105	4	30	4	300	2	30	2	30	180	30	Y	Y	Y	Y	Y	10	7	360	240	960	2880	198	4038	High	3	N
36	101	0.517	0	0	1	30	0	0	2	0	30	0	N	N	N	N	N	1	1	30	30	0	120	0	120	Low	1	N
37	77	0.060	0	0	7	60	6	20	5	0	60	20	N	N	N	Y	Y	13	7	80	80	0	1680	396	2076	Moderate	2	N
38	97	0.054	7	60	0	0	0	0	5	60	0	0	Y	Y	Y	N	Y	7	7	60	60	3360	0	0	3360	High	3	N
39	91	0.497	0	0	1	40	1	30	1.50	0	40	30	N	N	N	N	N	2	2	70	70	0	160	99	259	Low	1	N
40	103	0.064	0	0	1	180	5	15	4	0	180	15	N	N	N	N	Y	6	6	195	195	0	720	248	968	Moderate	2	N
41	156	0.634	0	0	0	0	5	30	5	0	0	30	N	N	N	Y	N	5	5	30	30	0	0	495	495	Moderate	2	N
42	89	0.325	0	0	5	180	1	15	1	0	180	15	N	N	N	Y	Y	6	6	195	195	0	3600	50	3650	Moderate	2	N
43	136	0.062	0	0	2	150	5	30	3	0	150	30	N	N	N	Y	Y	7	7	180	180	0	1200	495	1695	Moderate	2	N
44	84	0.057	0	0	3	120	5	20	2.50	0	120	20	N	N	N	N	Y	8	7	140	140	0	1440	330	1770	Moderate	2	N
45	79	0.554	0	0	0	0	1	60	0.50	0	0	60	N	N	N	N	N	1	1	60	60	0	0	198	198	Low	1	N
46	128	0.298	0	0	2	20	2	120	0.5	0	20	120	N	N	N	N	N	4	4	140	140	0	160	792	952	Low	1	N

CASE MDA	case MDA ng/ml	CASE IPAQ	CONTROL MDA	control MDA ng/ml	CONTROL IPAQ	CONTROL IPAQ quality	blood sugar case	blood sugar control	total colesterol case	trigcase	hdlcase	vldl case	ldl case	TGO case	TGP case	colestcont	trigcont	hdlcont	vldlcont	ldlcont	tgocont	tgpcont
0.563	46.83	720	0.074	5.56	198	1	85	99	186	292	54	58	74	32	54	154	42	57	8	89	20	26
0.822	71.43	2880	0.116	6.60	7200	3	94	86	226	153	46	31	149	15	16	208	72	60	14	134	25	25
0.626	54.39	399	0.060	6.69	2118	2	99	90	248	260	49	52	147	30	33	309	528*	108	-	-	23	32
0.536	46.57	179	0.066	11.29	1440	1	77	110	247	367	76	73	98	19	18	328	309	66	62	200	33	42
0.673	58.48	99	0.134	4.69	2628	1	93	91	206	123	51	25	130	23	19	202	92	49	18	135	13	16
0.552	47.96	198	0.141	5.21	4320	3	97	90	207	108	66	22	119	21	21	215	68	57	14	144	18	21
0.534	46.40	960	0.173	4.95	2373	2	110	98	207	88	68	18	121	26	36	136	149	37	30	69	25	28
0.451	39.19	876	0.151	5.38	1038	2	98	219	195	90	76	18	101	20	15	239	320	68	64	107	54	48
0.520	45.18	792	0.118	13.12	1995	2	89	99	225	132	48	26	151	27	26	174	83	54	17	103	22	22
0.635	55.18	4485	0.138	11.99	792	2	90	96	199	135	78	27	94	22	24	252	380	100	76	76	20	40
0.531	46.14	3360	0.082	10.25	0	1	103	97	193	119	50	24	119	23	27	237	284	78	57	102	26	31
0.543	47.18	707	0.187	11.29	3012	1	97	109	154	241	40	48	66	23	29	192	70	56	14	122	15	17
0.514	44.66	5235	0.087	12.25	1417	2	90	71	228	173	63	35	130	21	12	143	85	52	17	74	28	24
0.517	36.23	120	0.128	5.90	4665	3	101	91	231	192	59	38	134	20	14	183	144	62	29	92	19	18
0.497	43.18	259	0.130	8.51	579	1	91	86	158	58	40	12	106	30	21	177	211	57	42	78	27	25
0.634	55.09	495	0.098	7.56	200	1	156	73	167	73	62	15	90	151	215	184	132	65	26	93	25	32
0.554	48.14	198	0.076	13.20	231	1	79	76	158	88	49	18	91	21	17	142	108	44	22	76	35	61
0.298	25.89	952	0.152	11.55	1638	1	128	89	173	270	62	54	57	19	11	148	118	50	24	74	24	19
	5.56	198	0.068	7.50	1980	2		81								215	87	49	17	149	34	58
	6.60	7200	0.077	9.12	2730	2		328								189	92	67	18	104	59	70
	6.69	2118	0.133	5.90	1158	2		80								127	128	44	26	57	19	25
	11.29	1440	0.105	15.03	4038	3		76								190	266	63	53	74	29	29
	4.69	2628	0.060	28.24	2076	2		77								176	214	59	43	74	23	23
	5.21	4320	0.054	11.12	3360	3		97								189	86	98	17	74	25	17
	4.95	2373	0.064	10.08	968	2		103								155	88	28	18	109	24	29
	5.38	1038	0.325	5.73	3650	2		89								232	139	56	28	148	37	21
	13.12	1995	0.062	7.12	1695	2		136														
	11.99	792	0.057	5.21	1770	2		84														
	10.25	0																				
	11.29	3012																				
	12.25	1417																				
	5.90	4665																				
	8.51	579																				
	7.56	200																				
	13.20	231																				
	11.55	1638																				
	7.50	1980																				
	9.12	2730																				
	5.90	1158																				
	15.03	4038																				
	28.24	2076																				
	11.12	3360																				
	10.08	968																				
	5.73	3650																				
	7.12	1695																				
	5.21	1770																				
